# An Update on Antitumor Efficacy of Catechins: From Molecular Mechanisms to Clinical Applications

**DOI:** 10.1002/fsn3.70169

**Published:** 2025-04-18

**Authors:** Patrick Valere Tsouh Fokou, Boniface Kamdem Pone, Regina Appiah‐Oppong, Vincent Ngouana, Issakou Bakarnga‐Via, David Ntieche Woutouoba, Valerie Flore Donfack Donkeng, Lauve Rachel Tchokouaha Yamthe, Fabrice Fekam Boyom, Dilek Arslan Ateşşahin, Javad Sharifi‐Rad, Daniela Calina

**Affiliations:** ^1^ Department of Biochemistry, Faculty of Science University of Bamenda Bambili Cameroon; ^2^ Department of Biochemistry, Faculty of Science University of Yaounde 1 Yaounde Cameroon; ^3^ Department of Clinical Pathology, Noguchi Memorial Institute for Medical Research, College of Health Sciences University of Ghana Accra Ghana; ^4^ Department of Pharmaceutical Sciences, Faculty of Medicine and Pharmaceutical Sciences University of Dschang Dschang Cameroon; ^5^ Department of Biomedical and Pharmaceutical Sciences, Faculty of Human Health Sciences University of Ndjamena Ndjamena Chad; ^6^ Centre Pasteur du Cameroun Yaounde Cameroon; ^7^ Institute of Medical Research and Medicinal Plants Studies (IMPM) Yaoundé Cameroon; ^8^ Department of Plant and Animal Production, Baskil Vocational School Fırat University Elazıg Turkey; ^9^ Universidad Espíritu Santo Samborondón Ecuador; ^10^ Centro de Estudios Tecnológicos y Universitarios del Golfo Veracruz Mexico; ^11^ Department of Medicine, College of Medicine Korea University Seoul Republic of Korea; ^12^ Department of Clinical Pharmacy University of Medicine and Pharmacy of Craiova Craiova Romania

**Keywords:** catechins, chemoprevention, molecular mechanisms, oncology, pharmacodynamics, polyphenols, therapeutics

## Abstract

Carcinogenesis is the process by which substances that cause cancer (carcinogens) produce cancer. Extensive research conducted in recent years shows that the risk of developing certain cancers can be reduced by eating a variety of fruits and vegetables regularly. Catechins, which are more prevalent in foods and beverages made from plants, are known to have anti‐cancer effects. Detailed mechanistic studies are helpful in understanding the inhibitory effects of catechins on carcinogenesis and providing background information for evaluating the effects of catechins on human carcinogenesis. This article provides an overview of catechins and their potential roles in cancer prevention and treatment, focusing on how they alter signaling pathways, slow cell proliferation, and trigger apoptosis. Also, this article discusses molecular modifications of epigallocatechin gallate and catechins as well as delivery methods based on nanostructures.

## Graphical abstract

This article explores the potential of catechins, particularly (−)‐epigallocatechin‐3‐gallate (EGCG) as natural compounds with anticancer effects. Catechins, abundant in plants like green tea, have shown promise in modulating key processes in carcinogenesis, including apoptosis, cell cycle arrest, and angiogenesis. The review emphasizes their potential in cancer prevention and treatment, detailing how they alter signaling pathways and discussing molecular modifications of EGCG and catechins. The challenges faced by catechins, such as low bioavailability, variable pharmacokinetics, and the need for standardized formulations, are discussed. Despite promising in vitro and in vivo results, the translation into clinical practice is limited, and the article suggests future research directions to optimize catechin delivery, explore synergies with existing anticancer drugs, and address challenges for effective clinical application.

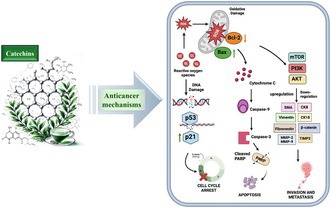

**Abbreviations**
5‐8F: nasopharyngeal carcinoma cells
A549: adenocarcinomic human alveolar basal epithelial cells
Akt (PKB): protein kinase B
AR: androgen receptor
ATP: adenosine triphosphate
B[a]P: benzo[a]pyrene
Bax: Bcl‐2‐associated X protein
Bcl‐2: B‐cell lymphoma‐2
Bcl‐x(L): extra‐large B‐cell lymphoma
Caco2: colon adenocarcinoma
CD44: cell surface adhesion receptor
cdk: cyclin‐cyclin‐dependent kinase
CDK4: cyclin dependent kinase 4
cki: cyclin kinase inhibitor
C‐Myc: MYC proto‐oncogene, BHLH transcription factor
CNE‐2: nasopharyngeal carcinoma cells
COX‐2: cyclooxygenase‐2
CSCs: human cancer stem cells
DNA: deoxyribonucleic acid
EGCG: epigallocatechin‐3‐O‐gallate
EMP: epithelial‐mesenchymal transition
ERK: extracellular signal‐regulated kinase
GADD: growth arrest and DNA damage‐inducible
GSK‐3β: glycogen synthase kinase‐3 β
Hs578T: human breast cancer epithelial cells
HT‐1080: fibrosarcoma cells
HuH7: hepatocyte derived cellular carcinoma cells
IFN: interferon
IGF‐1: insulin‐like growth factor
IGF‐1R: insulin‐like growth factor 1 receptor
iNOS: inducible nitric oxide synthase
LNCaP: androgen‐sensitive human prostate adenocarcinoma cells
MAPK: mitogen‐activated protein kinase
MCF 10A: non‐malignant breast epithelial cells
MCF‐7: breast cancer derived cells
MDA‐MB‐231: metastatic mammary adenocarcinoma 1
mdm2: mouse double minute 2 homolog
MMP‐2: matrix metalloproteinase‐2
MMP‐9: matrix metalloproteinase‐9
NF‐kB: nuclear factor kappa B
OVCAR‐3: human ovarian cancer cells
p21: cyclin‐dependent kinase inhibitor 1
PA‐1: human ovarian teratocarcinoma cell line
p‐Akt: phosphorylated Akt
PCNA: proliferating cell nuclear antigen
PDX: patient‐derived xenografts
p‐ERK 1/2: extracellular signal‐regulated kinase
p‐GSK‐3β: phospho‐glycogen synthase kinase‐3 β
p‐IGF‐IR: phospho‐insulin‐like growth factor receptor
PLGA: poly(lactic‐co‐glycolic acid)
PTEN: phosphatase and tensin homolog
Rb: retinoblastoma protein
RNA: ribonucleic acid
SD: Sprague–Dawley rats
SK‐OV‐3: human ovarian cancer cells
snRNAs: small nuclear RNAs
Sox2: SRY (sex determining region Y)‐box 2
TP53: tumor protein p53
TRAMP: transgenic adenocarcinoma of the mouse prostate
VEGFR‐2: vascular endothelial growth factor receptor

## 1 Introduction

Cancers are classified according to the type of organ or tissue of origin, but increasingly to the molecular characteristics of the cancer cells (Krieghoff‐Henning et al. 2017). In 2019, an average of 23.6 million new instances of cancer with 10 million deaths was recorded worldwide, ensuing in nearly 250 million disability‐adjusted life years (Global Burden of Disease 2019 Cancer Collaboration 2022; Ferlay et al. 2019). If this trend is maintained, cancer will soon overtake heart disease as the leading cause of death in the world (Lin et al. 2021). Effective and community‐based cancer prevention and management interventions are critical in the future reduction of the global cancer burden (Lin et al. 2021). Because of recent technological advances, it is now possible to amass knowledge about the development and progression of cancer, which can be used to develop more effective and/or less toxic cancer therapies (Krieghoff‐Henning et al. 2017). Natural products with chemopreventive capacity against carcinogenic processes have gained popularity in chemotherapy for most cancers (Fu et al. 2024). Green tea is one of the most consumed aromatic beverages worldwide. Many investigations correlated tea consumption and a dose‐dependently reduced risk of various metabolic disorders and mortality (Shukla et al. 2018).

Catechins are a flavon‐3‐ols that represent the most abundant polyphenols found in a variety of medicinal plants, especially green tea, *Camellia sinensis*, and *Camellia assamica*. They constitute 30%–40% of the dry tea leaves and are considered as a cheaper, readily relevant, and secure phytochemicals (Cheng et al. 2020; Kumar et al. 2022). (−)‐Epicatechin‐3‐gallate, (−)‐epigallocatechin (EGC), (−)‐epigallocatechin‐3‐gallate (EGCG), and (−)‐epicatechin are the main green tea, catechins (Bae et al. 2020; Cheng et al. 2020; Kumar et al. 2022). Catechins have numerous advantages, including the ability to regulate metabolism and to prevent or reduce skin damage, oxidative stress, inflammation, and proliferation of cancer cells (Bae et al. 2020; Hung et al. 2022). Catechins and EGCG, in particular, have been shown to interfere with molecular pathways involved in carcinogenesis among the compounds studied thus far (Cheng et al. 2020; Kumar et al. 2022). Green tea catechin (GTC) activity has been linked to the oncoproteins including a number of cell cycle regulators, activator protein 1, androgen receptors, Bax, Bcl‐2, or Bcr‐Abl, epidermal growth factor receptor, matrix metalloproteinases (MMP‐2 and MMP‐9), and p27 (Kumar et al. 2022).

Despite numerous studies on catechins, a critical assessment of their anticancer mechanisms remains insufficiently explored. The aim of the study is to critically review and synthesize existing research on the chemical characteristics and pharmacological properties of catechins, with a focus on elucidating their mechanisms of action in cancer prevention and treatment.

## 2 Methodology

The methodology employed in this review followed a systematic and structured approach to identify, evaluate, and synthesize relevant literature on the anticancer properties of catechins. Extensive searches were conducted in PubMed/MedLine, Web of Science, Scopus, and Google Scholar. Articles published in English from January 2000 to December 2023 were considered.

Keywords related to catechins and cancer such as “catechins,” “cancer,” “antioxidant,” and “polyphenols,” were used for searching and the next Medical Subject Headings (MeSH) like “Catechin,” “Neoplasms,” and “Pharmacology” were utilized. Keywords and MeSH terms were combined using Boolean operators like “AND” and “OR” for comprehensive search results.

### 2.1 Inclusion Criteria

Peer‐reviewed original research articles and reviews detailing the pharmacological effects and mechanisms of catechins in cancer were included.

### 2.2 Exclusion Criteria

Non‐peer‐reviewed articles, studies not focusing on catechins, and duplicated studies were excluded. Information on the chemical structure, pharmacological properties, and anticancer mechanisms of catechins was extracted; the chemical structures of catechins mentioned in the selected articles were validated using the PubChem database (PubChem). The taxonomy of the plants producing catechins was verified using the World Flora Online database (WFO 2023). The findings were qualitatively synthesized, emphasizing pharmacological actions.

## 3 Catechins: An Overview of Their Natural Sources, Chemical Characterization, Synthesis and Semi‐Synthetic Derivatives

### 3.1 Natural Sources

*Acalypha wilkesiana* *Muell Arg*, *Camellia sinensis* , *Camellia assumica*, *Ficus cordata*, *Ficus gnaphalocarpa*, *Ficus mucuso*, *Guibourtia coleosperma* , *Holothuria atra* , *Khaya grandifoliola CDC*, *Peltophorum africanum*, *Psidium guajava* , *Terminalia sericea* , and *Thalassodendron ciliatum* are just a few examples of diverse plant species that contain catechins. However, green tea from *Camellia sinensis* remains the highest source of catechins, mainly EGCG. Fruits such as strawberries, kiwis, blueberries, apples, and grape seeds; vegetables such as green beans, broccoli, celery, kale, onions, lettuce, tomatoes, or seaweed; and beverages like red wine, beer, tea, red wine, liqueurs, chocolate, cocoa, etc., are all sources of catechins (Batista et al. 2021; Mbaveng et al. 2014; Shukla et al. 2018; Zanwar et al. 2014). However, they differ significantly in terms of content and nature between sources, which differ across the globe. Raw tea, apricots, broad beans, black grapes, strawberries, and wine are said to contain significant amounts of catechin. High levels of catechins have also been found in raspberries, blackberries, cherries, black grapes, chocolate, broad beans, pears, and apples (Batista et al. 2021; Shukla et al. 2018).

### 3.2 Structure and Chemical Properties of Catechins

Catechins belong to the group of flavonoids and the subgroup of flavan‐3‐ols, and the biosynthesis of catechins in plants involves two metabolic pathways: shikimic‐cinnamic acid and acetate‐malonate pathways (Aryal et al. 2024). Catechin is made up of two benzene rings named A and B and one heterocyclic dihydropyran ring named C, both of which have a hydroxyl group at carbon 3. Catechins occur either free or esterified and contain two chiral centers on carbons 2 and 3, allowing them to be either in trans like ((+)‐catechin or cis like ((−)‐epicatechin)) due to (Batista et al. 2021; Kopustinskiene et al. 2021).

The presence of 3‐OH group in catechins allowed rings A, B, and C to arrange planarly and connect with the B‐ring through a hydrogen bond. The hydroxyl group's location in the basic flavone structure's B and C rings varies between these catechins. The B ring's ortho‐dihydroxyl group contributes to the compound's ability to scavenge free radicals. The third position of the C ring's gallate moiety boosts the ability of the ring to scavenge radicals. Catechins are metabolized relatively quickly, and this metabolism involves chemical alterations. Catechol‐O‐methyltransferase, UDP‐glucuronosyltransferase, and phenol sulfotransferase are enzymes involved in the glucuronidation, O‐methylation, and sulfation reactions that occur during Phase II metabolism of catechins. The metabolites produced when catechins are broken down have a huge range of advantageous biological functions (Banerjee et al. 2021; Kopustinskiene et al. 2021).

Catechins are quantified using UV absorption. Major catechins in green tea EC ((−)‐epicatechin), ECG ((−)‐epicatechin gallate), EGC ((−)‐epigallocatechin), EGCG ((−)‐epigallocatechin gallate) absorb at 210 or 269–280 nm. Condensed tannins, also called proanthocyanins, are oligomers and polymers of flavan‐3‐ol moieties, most commonly (−)‐epicatechin or (+)‐catechin. They are mostly present in grape seeds and wine, are created during catechin polymerization, and can turn into colorful anthocyanidins in hot, acidic environments (Bae et al. 2020; Batista et al. 2021; Kopustinskiene et al. 2021). They have many chemical structural capabilities, such as hydroxyl groups (−OH), that integrate effortlessly with various other constituents.

**FIGURE 1:**
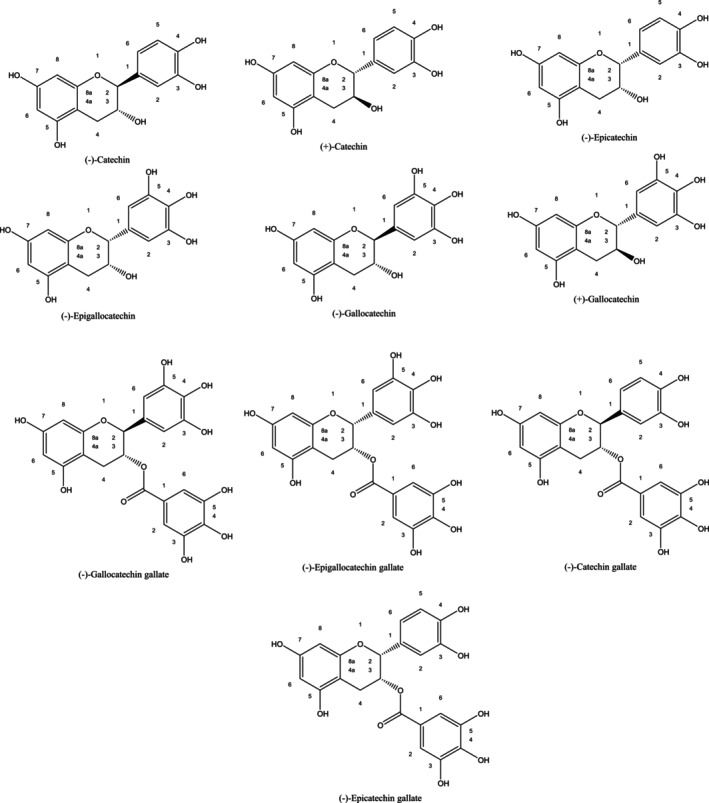
Chemical structures of most encountered catechins.

There are many types of catechins, but the most common catechins are (−)‐epigallocatechin, (+)‐gallocatechin, (+)‐catechin, and (−)‐epicatechin, which also exist as gallate bodies. Increase (Figure 1): ((−)‐catechin), EC ((−)‐epicatechin), ECG ((−)‐epicatechin gallate), EGC ((−)‐epigallocatechin), EGCG ((−)‐epigallocatechin gallate), GC ((−)‐gallocatechin), CG ((−)‐catechin gallate), and GCG ((−)‐gallocatechin gallate) (Bae et al. 2020; Spizzirri et al. 2019).

### 3.3 Semi‐Synthetic Derivatives

Catechins are not very stable and are easily oxidized (Muhammad and Dickinson 2019). Catechins are sensitive to pH and temperature and are changed by exposure to heat, especially during the processing of food (Chaudhary et al. 2023). For instance, heat‐treated catechins are degraded and epimerized into epicatechin. In fact, a study found that 36% of catechin was reduced when heated for 30 min at 100°C, with 9.7% converted to epicatechin. Additionally, it was discovered that sugars modulate catechins degradation. For instance, trehalose was found to inhibit catechins degradation while sucrose promotes it. 10% of sucrose caused degradation of 48% of catechin, with 22% epimerizing to epicatechin. Concerning the pH effect, it was found that (−)‐epigallocatechin and (−)‐EGCG were entirely degraded after 3 and 6 h, respectively, at pH 7.4 and 100°C. Nevertheless, degradation was greatly lessened at lower pH ranges (5–6.5). Green tea extract was found to degrade epigallocatechin and EGCG by 70% and 79%, respectively, when used at a concentration of 0.3%. Tea polyphenols started to degrade after building up in baked goods like cakes, biscuits, and bread. For bread containing 150 mg/100 g catechins, only 18.4% remained after baking at 215°C for 11 min. Thermal treatment can cause catechins such as epicatechin gallate, catechin gallate, EGCG, gallocatechin, and epigallocatechin to break down into different compounds like epicatechin, catechin, or gallic acid. (+)‐Catechin, (−)‐epicatechin, and their gallate conjugates, (+)‐gallocatechin (GC), and (+)‐catechin gallate (CG) are all monomeric catechins that can bind together and form dimers or disintegrate into protocatechuic acid and 3,4‐dihydroxybenzaldehyde (Kopustinskiene et al. 2021; Ou 2021). The permethylation of the catechin derivatives, EC, ECG, EGC, and EGCG, with potassium carbonate and dimethyl sulfate in refluxing anhydrous acetone produces a 90% overall yield. EGCG was used to create trimethylated and pentamethylated EGCG (Wang et al. 2017).

## 4 Mechanistic Insights Into the Anticancer Properties of Catechins

### 4.1 Anticancer Mechanisms of Catechins: Targeting the Hallmarks of Cancer

Carcinogenesis is the development of cancerous cells, often as a result of exposure to carcinogens (Buga et al. 2019; Cunha et al. 2023; Zlatian et al. 2015). Catechins, like (−)‐epigallocatechin‐3‐gallate, inhibit cell growth and induce cell death, making them a potentially valuable tool in cancer prevention. Many studies have been carried out to unveil the underlying anticancer mechanism of action of catechins (Bae et al. 2020; Sari 2019; Yu et al. 2014). The anticancer actions of catechins, specifically EGCG, align with multiple hallmarks of cancer, including modulating oxidative stress, cell cycle arrest, apoptosis, angiogenesis inhibition, signal transduction pathway modulation, epigenetic changes, and metastasis suppression. These multifaceted mechanisms underscore the potential of catechins as anticancer agents.

#### 4.1.1 Antioxidant Activity and Oxidative Stress Modulation

The interplay between antioxidant activity and oxidative stress modulation is a cornerstone in understanding the anticancer properties of catechins (Bernatoniene and Kopustinskiene 2018; GBD 2019 IMID Collaborators 2023; Zhao et al. 2023). Catechins, particularly EGCG, possess strong free radical‐scavenging activity due to their hydroxyl‐rich chemical structure, which enables them to neutralize reactive oxygen species (ROS) and reduce oxidative damage to cellular components, including lipids, proteins, and DNA. These natural compounds, prevalent in green tea, have been shown to function as potent antioxidants, providing a protective shield against oxidative stress, a critical contributor to the carcinogenesis process (Bernatoniene and Kopustinskiene 2018). Oxidative stress, characterized by an imbalance between free radicals and antioxidants in the body, can lead to cellular damage, DNA mutations, and, ultimately, cancer development (Sharifi‐Rad et al. 2022; Zhao et al. 2023). Catechins mitigate this imbalance by upregulating endogenous antioxidant defense mechanisms, such as the expression of superoxide dismutase (SOD) and glutathione peroxidase (GPx), while downregulating ROS‐generating enzymes like NADPH oxidase and through their strong antioxidative actions, help maintain this delicate balance, thereby mitigating the risk of cancer initiation and progression (Chaudhary et al. 2023). Additionally, catechins influence critical pathways involved in oxidative stress response, including the activation of Nrf2 (nuclear factor erythroid 2‐related factor 2), a key transcription factor that regulates antioxidant response elements (AREs) and promotes cellular resistance to oxidative damage. Recent studies shown to work as a strong antioxidant that protect cells against oxidative stress, a modulator of angiogenesis and tumor cell response through apoptosis by increasing caspases (Iwasaki et al. 2009) and by altering the expression of cell cycle regulatory proteins (CYC/Cdk) (Khiewkamrop et al. 2022). These multifaceted antioxidant mechanisms underscore the therapeutic potential of catechins in cancer prevention and treatment.

#### 4.1.2 Apoptosis Induction and Cell Cycle Arrest

Natural compounds such as catechins induce apoptosis and regulate cell cycle arrest by modulating key molecular pathways, as observed with other promising phytochemicals like diosmin, betulinic acid, and alpha‐solanine, which exhibit similar anticancer mechanisms including caspase activation and cell cycle checkpoint regulation (Nandi et al. 2024; Nemli et al. 2024; Rahman et al. 2024). The utmost prevalent and chief cause of death for women is breast cancer. The catechin hydrate inhibits the growth of MCF‐7 cells and induces apoptosis; this is thought to be due to its ability to increase the expression of pro‐apoptotic genes, for instance caspase‐3, caspase‐8, and caspase‐9, as well as TP53 (Alshatwi 2010). In sarcoma 180 cells, EGCG controls the G2/M phase of the cell cycle by upregulating p53 and Bax while downregulating C‐Myc, Bcl‐2, and spliceosomal uridylate‐rich small nuclear RNA (UsnRNA), U1B, and U4‐U6 (Manna et al. 2006). EGCG inhibits ovarian cancer cell proliferation by apoptosis and cell cycle arrest in G(1) phase in SKOV‐3 and OVCAR‐3 cells, G(1)/S arrest in PA‐1 cells, and cell cycle‐associated proteins. EGCG seems to have a gene regulatory role, based on how it differentially regulates the gene expression of various proteins (Bcl‐x(L), Bax, p21, cyclin D1, CDK4). This suggests that EGCG might act like p53 proteins when it comes to facilitating apoptosis. Additionally, Bcl‐x, Bax, and PCNA appear to be important in EGCG‐mediated apoptosis. However, CDK4 and retinoblastoma protein (Rb) do not seem to be as important in the inhibition of ovarian cancer cells (Huh et al. 2004). EGCG caused apoptosis by increasing caspase activity and blocked cells in the sub‐G1 phase of the cell cycle. Cd133 and abcg2, as well as other genes associated with stem cells and those linked to the clinical aggressiveness of the tumor, were also down‐regulated by EGCG (Mayr et al. 2015). EGCG previously showed to cause cell cycle arrest and death in human melanoma cells via altering the Bcl‐2 family proteins and cki‐cyclin‐cdk machinery. Further research revealed that EGCG could increase IFN's anti‐proliferative effects on melanoma, both in culture and in an animal model, via activating Fas and NF‐B signaling (Nihal et al. 2009).

#### 4.1.3 Modulation of Signal Transduction Pathways

Catechin can stop the cell cycle in G/S transition through the initiation of p27 expression in breast cancer; EGCG decreased the proliferation of the estrogen receptor‐negative breast cancer cell lines Hs578T and MDA‐MB‐231 in culture. The activation of the ectopic expression of the protein p27 (Kip1) CKI is one of the mechanisms through which EGCG, in culture, inhibited the growth of human breast cancer cells and carcinogen‐induced mammary tumorigenesis in female SD rats (Kavanagh et al. 2001). Both pI3K and the mammalian target of rapamycin (mTOR) are inhibited by EGCG. The latter inhibits cell proliferation of MDA‐MB‐231 and A549 cells by inducing AKT phosphorylation. EGCG efficiently binds to the active site of the PI3K kinase domain and competes for ATP binding in silico (Van Aller et al. 2011).

#### 4.1.4 Epigenetic Modifications and Gene Expression

Catechins, particularly EGCG, have been shown to influence epigenetic mechanisms, which are crucial in regulating gene expression and maintaining cellular homeostasis. EGCG causes DNA fragmentation and cell cycle arrest in the G(0)/G(1) phase, correlating with the induction of apoptosis via activation of p53 and caspases 7 and 9, and inhibition of p38MAPK activity, Bcl‐2, and phosphorylation of ERK 1 and 2, and other extracellular signal‐regulated protein kinases (ERKs) as well as NF‐kB (phosphorylated nuclear factor‐κB) (Lee et al. 2011; Maeda‐Yamamoto et al. 2003). Human skin cancer cells treated with EGCG showed increased histone acetylation on lysines of histone H3 and H4 that decreased DNA methylation, leading to upregulation of Cip1/p21 and p16INK4a, two tumor suppressor genes (Nandakumar et al. 2011). Treatment with EGCG dose‐dependently inhibits cell growth of human fibrosarcoma cells such as HT‐1080 cells. This inhibition has been linked to the disruption of signaling pathways that relay external growth signals to cell cycle and proliferation‐related genes, further emphasizing the role of catechins in altering epigenetic and gene expression dynamics (Lee et al. 2011). These findings highlight EGCG's potential as a natural epigenetic modulator that targets critical processes in cancer development, offering a novel avenue for therapeutic interventions.

#### 4.1.5 Inhibition of Angiogenesis

Hepatocellular carcinoma is a type of cancer that affects the liver, and one of the main ways that this cancer grows and spreads is through a process called angiogenesis. Green tea and (−)‐EGCG are effective in slowing the growth of this type of cancer by preventing the activation of certain types of proteins that are needed for cancer cell growth. In one study, it was shown that green tea was able to time‐ and dose‐dependently reduce the growth of HuH7 human hepatocarcinoma cells by preventing the expression of certain proteins (p‐VEGFR‐2 and VEGFR‐2) that are needed for angiogenesis. This decrease in activity of downstream signaling molecules like VEGFR‐2 and VEGF, ERK, Akt, as well as Bcl‐x(L) was also observed in HuH7 xenografts in nude mice (Shirakami et al. 2009). EGCG inhibited the growth and proliferation of nasopharyngeal carcinoma (CNE‐2 and 5‐8F) cells by inducing apoptosis (cell death) through the downregulation of an NAD+‐dependent protein deacetylase, sirtuin 1 (SIRT1) that plays an important role in cancer cell metabolism (Jiang et al. 2022). Green tea inhibits LNCaP cell growth by inducing apoptosis, suppressing tumor growth, and upregulating miR‐181a expression (Safari et al. 2022). It was discovered that apoptosis and cell cycle arrest caused by EGCG involve a complex interaction with the cki‐cdk machinery. In androgen‐sensitive LNCaP and androgen‐insensitive DU145 human prostate carcinoma cells, upregulation of the protein expression of INK4c/p18, INK4a/p16, KIP1/p27, and WAF1/p21, and downmodulation of cdk 2, 4, and 6, cyclin D1, and cyclin E result in programmed cell death. Cell cycle arrest at the G1 phase is caused by reducing the binding of cyclin E to cdk2 and increasing the binding of cyclin D1 to WAF1/p21 and KIP1/p27, which inactivates the cyclin‐cdk complexes active in the G0/G1 phase of the cell cycle and induces apoptosis in human prostate carcinoma cells [51]. In TRAMP mice prostate cancer, catechins suppress cell growth and induce cell apoptosis by reducing AR, COX‐2, IGF‐1, IGF‐1R, iNOS, and p‐ERK 1/2, showing suppression of early‐stage tumors (Cheng et al. 2020). In mouse models of colorectal tumors, catechin treatment reduced the expression of β‐catenin, cyclooxygenase‐2, IGF‐IR (p‐IGF‐IR), p‐GSK‐3β, and cyclin D1 proteins, resulting in the inhibition of the development of malignant intestinal lesions (Shimizu, Shirakami, Sakai, Adachi, et al., 2008). Epicatechin gallate and EGCG have regulatory effects on benzo[a]pyrene (B[a]P)‐induced lung cancer in mice through differential regulation of p53 and expression of its associated genes, including p21 and 27, Bcl‐2, bax, mdm2, C‐Myc, H‐ras, and cyclin D1 (Manna et al. 2009).

#### 4.1.6 Inhibition of Metastatic Processes

**FIGURE 2:**
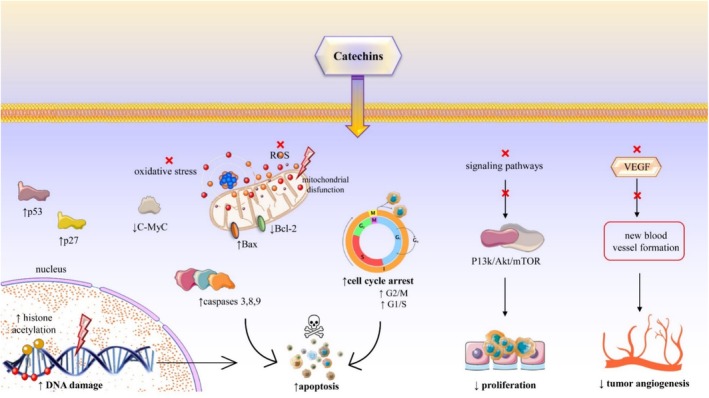
Summarized diagram regarding mechanisms by which catechins exert antineoplastic effects on cancer cells. The figure depicts an increase in p53 and p27 protein levels, leading to DNA damage and cell cycle arrest at the G2/M and G1/S checkpoints. Concurrently, catechins inhibit C‐Myc, Bcl‐2, and VEGF, which reduces oxidative stress, mitochondrial dysfunction, and apoptosis reactively. The downregulation of the PI3k/Akt/mTOR pathway results in decreased cell proliferation. Furthermore, increased levels of Bax and caspases 3, 8, and 9 induce apoptosis in cancer cells. Akt, protein kinase B; Bax, Bcl‐2‐associated X protein; Bcl‐2, B‐cell lymphoma 2; c‐Myc, Myc proto‐oncogene protein; mTOR, mammalian target of rapamycin; PI3k, phosphoinositide 3‐kinase; ROS, reactive oxygen species; VEGF, vascular endothelial growth factor. Symbols: ↑, increase; ↓, decrease; x, inhibition.

Human cancer stem cells (CSCs) are a known targets of EGCG for cancer prevention and treatment; the fact that EGCG prevents the transcription and translation of genes encoding for stemness markers such as CD44, CD133, Nanog, Oct4, and Sox2 suggests that it averts CSCs from self‐renewing in general (Fujiki et al. 2017). The epithelial‐mesenchymal transition markers including Twist, Snail, vimentin, and aldehyde dehydrogenase of human CSCs are also suppressed by EGCG. This compound had a smaller effect on CSC stemness than it did on parental cells. When used in conjunction with anticancer medications, EGCG's meager inhibitory action was strengthened synergistically (Fujiki et al. 2017). Table 1 and Figure 2 summarize the mechanisms through which catechins act against different types of cancers.

**TABLE 1:** Anticancer mechanisms of catechins, molecular targets, and cellular impact.

Anticancer mechanism	Molecular targets and cellular effects	References
Antioxidant Oxidative stress modulation	↑ Antioxidant protection ↓ Oxidative stress ↓ Angiogenesis and tumor cell response through apoptosis by ↑ caspases Alters the expression of cell cycle regulatory proteins (cyc/cdk)	Iwasaki et al. (2009), Khiewkamrop et al. (2022)
Apoptosis induction	↑ Caspase‐3, caspase‐8, caspase‐9 expression ↑ TP53 expression and apoptosis in MCF‐7 cells and sarcoma 180 cells ↑ Apoptosis in ovarian cancer cells (SKOV‐3, OVCAR‐3, PA‐1) and melanoma cells ↑ Caspase activity, ↑ cell cycle arrest	Alshatwi (2010)
Cell cycle arrest	↑ p53, ↑ Bax, ↓ C‐Myc, ↓ Bcl‐2 ↑ G2/M phase via UsnRNA regulation (U1B, U4‐U6) ↑ G(1)/S arrest	Huh et al. (2004), Manna et al. (2006)
Signal transduction pathway modulation	↑ p27 expression, ↓ cell proliferation ↓ pI3K, ↓ mTOR, ↑ Akt phosphorylation	Kavanagh et al. (2001), Van Aller et al. (2011)
Epigenetic modifications	↑ DNA fragmentation ↑ Histone acetylation ↓ DNA methylation	Lee et al. (2011), Maeda‐Yamamoto et al. (2003), Nandakumar et al. (2011)
Angiogenesis inhibition	↓ VEGFR‐2 expression Prevents angiogenesis in HuH7 xenografts in nude mice	Shirakami et al. (2009)
SIRT1 downregulation	↑ Apoptosis by ↓ SIRT1 expression in nasopharyngeal carcinoma cells (CNE‐2, 5‐8F)	Jiang et al. (2022)
CSCs and EMT marker suppression	↓ Self‐renewal of CSCs ↓ Stemness markers (CD44, CD133, Nanog, Oct4, Sox2) ↓ EMT markers (Twist, Snail, Vimentin)	Fujiki et al. (2017)
Metastasis inhibition	↓ β‐catenin, ↓ COX‐2, ↓ p‐IGF‐IR, ↓ p‐GSK‐3β, ↓ cyclin D1 ↓ COX‐2, ↓ IGF‐1, ↓ IGF‐1R, ↓ iNOS, ↓ p‐ERK 1/2	Cheng et al. (2020), Manna et al. (2009), Shimizu, Shirakami, Sakai, Tatebe, et al. (2008)

*Note:* Symbols—↑, increase; ↓, decrease.

Abbreviations: Akt, protein kinase B; Bax, Bcl‐2‐associated X protein; Bcl‐2, B‐cell lymphoma 2; Bcl‐x(L), B‐cell lymphoma‐extra large; CD133, cluster of differentiation 133; CD44, cluster of differentiation 44; CDK4, cyclin‐dependent kinase 4; COX‐2, cyclooxygenase‐2; CSCs, cancer stem cells; Cyclin D1, cyclin D1; DNA, deoxyribonucleic acid; EMT, epithelial‐mesenchymal transition; ERK, extracellular signal‐regulated kinases; G(0)/G(1), Gap 0/Gap 1 phase; G2/M, Gap 2/mitosis phase; GSK‐3β, glycogen synthase kinase 3 beta; H‐ras, Harvey rat sarcoma viral oncogene; IGF‐IR, insulin‐like growth factor I receptor; mdm2, mouse double minute 2 homolog; Nanog, nanog homeobox; Oct4, octamer‐binding transcription factor 4; p16INK4a, cyclin‐dependent kinase inhibitor 2A; p21 (Cip1), cyclin‐dependent kinase inhibitor 1; p53 (TP53), tumor protein p53; p53‐like, referring to activities similar to those of TP53; p‐GSK‐3β, phosphorylated glycogen synthase kinase 3 beta; p‐IGF‐IR, phosphorylated insulin‐like growth factor i receptor; Rb, retinoblastoma protein; SIRT1, sirtuin 1; Snail, snail family transcriptional repressor; Sox2, SRY‐box transcription factor 2; Twist, Twist family BHLH transcription factor; UsnRNA, U‐small nuclear RNA; VEGFR‐2, vascular endothelial growth factor receptor 2.

### 4.2 Pharmacological Evidence of Anticancer Effects of Catechins

EGCG and other GTC are potent antioxidants found in green tea that showed anti‐cancer effects in various cell lines. The growth of human MCF‐7, MCF‐7/BOS, MDA‐MB‐231, and T47D MCF10A breast cancer cells was inhibited, and apoptosis was induced when exposed to EGCG at < 50 μM concentration (Baker and Bauer 2015; De Amicis et al. 2013; Huang et al. 2017; Luo, Wang, et al. 2010; Rathore et al. 2012). Catechin hydrate, a strong free radical scavenger, effectively kills MCF‐7 cells at 150 μg/mL through induction of apoptosis via increasing the expression of pro‐apoptotic genes, such as TP53, caspase‐3, caspase‐8, and caspase‐9 (Alshatwi 2010). Catechin inhibits the growth and migration of liver cancer cells at 20–100 μM in liver cancer cell lines, such as HepG2, Hep3B, HCC, HLE, HepG2, HuH‐7, and PLC/PRF/5 (Huang et al. 2009; Nishikawa et al. 2006; Shimizu, Shirakami, Sakai, Adachi, et al., 2008; Shimizu, Shirakami, Sakai, Tatebe, et al. 2008; Shirakami et al. 2009; Yang et al. 2019). In prostate cancer, catechin and EGCG inhibited cell growth, mitogenesis, and caused apoptosis at 1–80 μM in PC3, DU145, and LNCaP cells (Chuu et al. 2009; Duhon et al. 2010; Gupta et al. 2000, 2003; Hagen et al. 2013; Hastak et al. 2005; Luo, Luo, et al. 2010). EGCG inhibited the growth of colorectal cancer cells, such as HEK293, Caco2, HCT116, HT29, SW480, SW48, SW480, IEC6, and S W837 cell growth and induced cell apoptosis at 0–150 μM (Adachi et al. 2009; Cerezo‐Guisado et al. 2015; Morris et al. 2016; Oh et al. 2014; Shimizu and Weinstein 2005; Umeda et al. 2008). Furthermore, EGCG inhibited the growth of lung cancer cells, in addition to the spontaneous metastasis of melanoma cells. This compound can also suppress tumorigenesis and cell growth, and induce cell apoptosis at 20–100 μM. Green tea can induce ROS in H1299 and Lu99 cells, as well as H69, A549 cells, and CL13 and inhibits nicotine‐induced migration, invasion, and angiogenesis (Li et al. 2016; Sadava et al. 2007; Shi et al. 2015; Takahashi, Watanabe, et al. 2014, Takahashi, Kobayashi, et al. 2014). Among the tea catechins, particularly EGCG, have powerful anti‐cancer properties followed by ECG, EGC, and EC. Studies have shown that a combination of catechins is more active than pure EGCG in impeding tumor growth. When applied to human tumors, from breast, colon, esophagus, liver, lung, prostate, small intestine, and stomach, GTCs have an assortment of anti‐carcinogenic and anti‐mutagenic properties (Fujiki et al. 2015). Additionally, green tea extracts, tea catechin blends, or pure EGCG are able to influence the carcinogenesis process of tumor initiation, development, and advancement in genetically or chemically developed animal models of cancers (Singh et al. 2011). Most studies use cancerous cells from one species (xenografts) to study cancer growth in another species (like humans). This is done by injecting the cancerous cells subcutaneously (under the skin) into nude mice. Carcinogens are given orally or intraperitoneally in other models that are used in preclinical studies. Other models also used in preclinical investigations are achieved through oral or intraperitoneal administration of carcinogens. Typically, the tumors expand over time in proportion to the number of injected cells or the dose of carcinogens. Numerous investigational strategies were used to administer EGCG to mice through drinking water or food, oral gavage, and intraperitoneal injection, and revealed that catechin intake concentrations and treatment duration times varied between experiments (Cheng et al. 2020). Dextran‐catechin has shown to significantly reduce synergistically tumor growth in human neuroblastoma models in vivo, thus dextran‐catechin may be a valuable treatment against copper‐dependent cancers, such as neuroblastoma (Vittorio et al. 2016). Table 2 provides a summary of the anticancer effects of catechins, their inhibitory action on cell growth, and apoptosis induction across different cancer cell lines.

**TABLE 2:** Pharmacological evidence of anticancer effects of catechins.

Cancer type	Cell lines	Tested compound(s)	Concentration/dosage	Effects	References
Breast	MCF‐7 MCF‐7/BOS MDA‐MB‐231 T47D	EGCG	< 50 μM	↓ Growth ↑ Apoptosis	Baker and Bauer (2015), De Amicis et al. (2013), Huang et al. (2017), Luo, Wang, et al. (2010), Rathore et al. (2012)
	MCF‐7	Catechin Hydrate	150 μg/mL	↑ Apoptosis ↑ Pro‐apoptotic gene expression	Alshatwi (2010)
Liver	HepG2, Hep3B, HCC, HLE, HuH‐7, PLC/PRF/5	Catechin	20–100 μM	↓ Growth ↓ Migration inhibition	Huang et al. (2009), Nishikawa et al. (2006), Shimizu, Shirakami, Sakai, Adachi, et al. (2008), Shimizu, Shirakami, Sakai, Tatebe, et al. (2008), Shirakami et al. (2009), Yang et al. (2019)
Prostate	PC3, DU145, LNCaP	Catechin, EGCG	1–80 μM	↓ Cell growth ↓ Apoptosis	Chuu et al. (2009), Duhon et al. (2010), Gupta et al. (2000), Gupta et al. (2003), Hagen et al. (2013), Hastak et al. (2005), Luo, Luo, et al. (2010)
Colorectal	HEK293, Caco2, HCT116, HT29, SW480, SW48, SW837	EGCG	150 μM	↓ Growth ↑ Apoptosis	Adachi et al. (2009), Cerezo‐Guisado et al. (2015), Morris et al. (2016), Oh et al. (2014), Shimizu and Weinstein (2005), Umeda et al. (2008)
Lung	H1299, Lu99, H69, A549, CL13	EGCG	20–100 μM	↓ Tumorigenesis ↑ Apoptosis	Li et al. (2016), Sadava et al. (2007), Shi et al. (2015), Takahashi, Watanabe, et al. (2014), Takahashi, Kobayashi, et al. (2014)
Neuroblastoma	Human neuroblastoma mouse model	Dextran‐catechin	150–300 μg/mL	↓ Tumor growth	Vittorio et al. (2016)

Abbreviations: EGCG, epigallocatechin gallate; HCC, hepatocellular carcinoma; HEK, human embryonic kidney; HT29, human colon adenocarcinoma cell line; MCF, Michigan Cancer Foundation; PC3, DU145, LNCaP, prostate cancer cell lines; ROS, reactive oxygen species.

## 5 Advancements in the Pharmacokinetics, Bioavailability and Nanodelivery of Catechins: Enhancing Therapeutic Potential

### 5.1 Pharmacokinetics and Bioavailability of Catechins

Catechins, including EGCG, epicatechin gallate (ECG), epigallocatechin (EGC), and epicatechin (EC), are noteworthy for their health‐promoting properties, attributed to their antioxidative, anti‐inflammatory, and anticarcinogenic actions (Chaudhary et al. 2023). The pharmacokinetics of catechins, which include their absorption, distribution, metabolism, and elimination, critically influence their bioavailability and, consequently, their therapeutic effectiveness (Cai et al. 2018). Catechins are absorbed primarily in the small intestine, with factors such as pH and food matrix affecting their solubility and, therefore, their absorption rate (Koo and Cho 2004; Lippolis et al. 2023). EGCG, the most studied catechin, demonstrates relatively low oral bioavailability, partly due to its instability in the alkaline pH of the intestine and its extensive conjugation during first‐pass metabolism (Koo and Cho 2004). Following absorption, catechins are distributed throughout the body, with varying affinities for different tissues. Studies have shown preferential accumulation of certain catechins in organs like the liver, kidney, and intestine, which are crucial sites for metabolism and excretion (Cai et al. 2018). Catechins undergo extensive metabolism primarily in the liver and intestines; this involves Phase II biotransformation reactions, including methylation, sulfation, and glucuronidation (Feng 2006). The resultant metabolites often have altered biological activities and stability compared to the parent compounds (Cai et al. 2018; Feng 2006). Catechins and their metabolites are primarily excreted via the kidneys. The elimination half‐life of catechins varies, with EGCG having a half‐life of about 5–6 h (Lee et al. 2002). The excretion rate is influenced by various factors, including the specific catechin and its metabolites, as well as individual differences in metabolism (Lee et al. 2002). The bioavailability of catechins is influenced by intrinsic factors like molecular structure and stability, as well as extrinsic factors like food matrix and interaction with other dietary components (Cai et al. 2018). The presence of other polyphenols, fiber, and certain minerals and proteins can significantly impact the absorption and metabolism of catechins (Cai et al. 2018). Understanding and improving the bioavailability of catechins is fundamental for maximizing their therapeutic potential; low bioavailability may necessitate higher doses to achieve desired effects, but this must be balanced against the risk of potential toxicity and side effects (Cerbin‐Koczorowska et al. 2021).

### 5.2 Nanotechnology‐Enhanced Delivery Systems for Catechins: Enhancing Anticancer Efficacy

Catechins' stability, bioavailability, and biological characteristics have all been shown to be improved by nanodelivery, which also has the potential to increase the anticancer activity of this class of compounds and increase their oral bioavailability (Jiang et al. 2021). Strategies to enhance the bioavailability of catechins include the use of adjuvants like piperine, formulation modifications like nanoparticles or liposomes, and the co‐administration with other compounds that inhibit catechin metabolism (Cerbin‐Koczorowska et al. 2021). Carriers mainly lipid‐based and polymer‐based nanoparticles (NPs) varying from nanometers to micrometers were established to encapsulate and deliver catechins (Jiang et al. 2021). Generally speaking, natural polymer nanoparticles and synthetic polymer nanoparticles can be used in polymer‐based nanodelivery systems. The most common lipid‐based catechin delivery technique is solid lipid nanoparticles (SLNs), which are extremely plentiful. SLNs greatly increase the oral bioavailability of EGCG when compared to free EGCG, in rats with a significant increase in tissue such as liver, kidney, brain, and spleen. The kidney had the highest concentration of EGCG, whereas the spleen had the highest quantity of free EGCG. The oral administration of SLNs containing EGCG did not show any signs of acute or subchronic toxicity (Jiang et al. 2021). Nanoparticles have been synthesized using, β‐lactoglobulin, gelatin, hordein, chitosan‐tripolyphosphate complex, chitosan‐tripolyphosphate‐polyethylene glycol‐folate complex, chitosan‐β‐lactoglobulin complex, chitosan‐gelatin complex, β‐lactoglobulin‐gum arabic complex, ovalbumin‐dextran conjugate, chitosan‐coated bovine serum albumin, poly‐ε‐lysine‐ or chitosan‐coated bovine serum albumin, folate conjugated chitosan, chitosan‐polyaspartic acid, chitosan‐γ‐pga (poly(γ‐glutamic acid)), hyaluronic acid‐fucoidan‐polyethylene glycol‐gelatin, SLNs, nanoliposome, chitosan‐coated nanoliposome, nanostructured lipid carriers, chitosan‐coated nanostructured lipid carriers, dimeric c(RGD) peptide conjugated nanostructured lipid carriers, and nanoethosomes (Jiang et al. 2021). Catechins have also been encapsulated in proteins like gelatin, hordein, and lactoglobulin, showing a variety of enhancements that may help to increase their anticancer effects (Jiang et al. 2021). Combining EGCG and heat‐modified β‐lactoglobulin (β‐LG) to form stable co‐assembled nanoparticles (Eβ‐NPs) or EGCG‐loaded β‐lactoglobulin nanoparticles has been shown to be active in numerous types of cancer cells, particularly human melanoma A375 and esophageal carcinoma TE‐1 cells. These nanoparticles enhanced EGCG anticancer effects by suppressing proliferation, increasing apoptosis, and substantially arresting the cell cycle (Wu et al. 2017). Another class of natural polymers called polysaccharides, which include chitosan, alginate, arabic gum, and cyclodextrin, are utilized to nanoencapsulate and distribute catechins (Jiang et al. 2021). EGCG loaded with folate‐modified chitosan nanoparticles upregulates Bax, PTEN, p21, and downregulates cyclin D1, Bcl‐2, p‐PDK1, and p‐AKT, thereby reducing growth human breast cancer cells MCF‐7 (Zeng et al. 2017). Catechins are frequently enclosed in complicated coacervates using a combination of proteins and polysaccharides. EGCG‐loaded chitosan and ‐lactoglobulin complex nanoparticles improved EGCG's in vitro cellular antioxidant activity (Dai et al. 2020). Genipin cross‐linking displayed strong cytotoxicity against cancer cells and reduced the burst release of EGCG from nanoparticles (Kumar et al. 2016). EGCG nanoparticles (lecithin‐based and Poly (lactic‐co‐glycolic acid)‐based) showed superior anticancer activity compared to free EGCG in inhibiting lung cancer tumors in the PDX model by inhibiting NF‐κB activation and suppressing the expression of NF‐κB‐regulated genes (Zhang et al. 2020). Moreover, the growth of H1299 lung cancer cells was suppressed by free EGCG and its nanoemulsion form (nano‐EGCG), with IC_50_ of 36.03 and 4.71 M, respectively. Additionally, nano‐EGCG dose‐dependently inhibited lung cancer cell colonization, migration, and invasion by triggering the expression of numerous important AMPK signaling pathway regulatory proteins such as MMP‐2 and MMP‐9 (Chen et al. 2020). The pharmacokinetics and bioavailability of catechins are complex and influenced by numerous factors. Enhanced understanding of these processes is vital for the development of effective catechin‐based therapies and their application in clinical settings. Future research should focus on strategies to improve bioavailability and the development of novel delivery systems to enhance the therapeutic efficacy of catechins. Table 3 summarizes the various nanotechnology applications for catechin delivery, focusing on their molecular mechanisms in anticancer activity, the models used in studies, and the comparative efficacy.

**TABLE 3:** Nanotechnology applications for catechin delivery.

Nano delivery system	Key features	Anticancer mechanism	Study model	Anticancer efficacy	References
Solid lipid nanoparticles (SLNs)	Lipid‐based encapsulation of catechins	↑ Bioavailability ↑ Distribution of EGCG	Rat model	↑ Concentration in liver, kidney, brain, spleen	Jiang et al. (2021)
Protein‐based nanoparticles	β‐Lactoglobulin Gelatin Hordein complexes	↑ Apoptosis ↑ Cell cycle arrest	Human melanoma A375 Esophageal carcinoma TE‐1 cells	↑ Anticancer effects of EGCG	Wu et al. (2017)
Chitosan‐based nanoparticles	Folate‐modified chitosan for EGCG delivery	↑ Bax, ↑ PTEN ↑ p21, ↓ Cyclin D1 ↓ Bcl‐2 ↓ p‐PDK1 ↓ p‐AKT	Human breast cancer cells MCF‐7	↓ Tumor cell growth	Zeng et al. (2017)
Nanostructured lipid carriers	Combines lipids and polymers for EGCG encapsulation	↓ NF‐κB activation ↓ Gene expression	Lung cancer PDX model, H1299 cell line	Superior efficacy compared to free EGCG	Zhang et al. (2020), Chen et al. (2020)

*Note:* Symbols—↑, increase; ↓, decrease.

Abbreviations: Bax, Bcl‐2‐associated X protein, a protein promoting apoptosis; Bcl‐2, B‐cell lymphoma 2, a protein inhibiting apoptosis; Cyclin D1, a protein involved in cell cycle regulation; EGCG, epigallocatechin gallate; NF‐κB, nuclear factor kappa‐light‐chain‐enhancer of activated B cells; NLCs, nanostructured lipid carriers; p21, a cyclin‐dependent kinase inhibitor that regulates the cell cycle; p‐AKT, phosphorylated protein kinase B, a key signaling protein in various cellular processes, including metabolism and survival; PDX, patient‐derived xenograft; p‐PDK1, phosphorylated 3‐phosphoinositide‐dependent protein kinase‐1, involved in cell survival signaling; PTEN, phosphatase and tensin homolog, a tumor suppressor gene; SLNs, solid lipid nanoparticles.

## 6 Synergistic Anticancer Effects of Catechins With Conventional Chemotherapy

The combined action of anticancer medications and (−)‐EGCG has been investigated, with an emphasis on the reduction of cell growth and activation of apoptosis. It showed that EGCG and sulindac particularly caused the upregulation of the p21 and GADD153 genes in PC‐9 lung cancer cells (Suganuma et al. 2011). Celecoxib combined with EGCG synergistically increased apoptosis and GADD153 gene expression in human non‐small cell lung cancer cells via activation of the MAPK signaling pathway (Suganuma et al. 2011). Catechin strongly inhibits cell proliferation not only via reduction of cyclin E1 expression and protein kinase (p‐Akt) phosphorylation, but also modulates cell cycle arrest or indirectly increases the expression of p21 and 27 signaling pathways in A549 cells (Sun et al. 2020). EGCG causes cell cycle arrest in biliary tract cancer cells and acts synergistically with cisplatin. When EGCG and cisplatin were used together, five cell lines responded synergistically, whereas two cell lines responded antagonistically. Additionally, EGCG decreased the expression of cell cycle‐related genes while increasing the expression of the apoptosis‐associated death receptor 5 and the cell cycle inhibitor p21 (Mayr et al. 2015). Researchers have documented the possibility of working synergistically with non‐steroidal anti‐inflammatory drugs (NSAIDs) and GTC to inhibit tumor growth in animals by triggering the GADD153‐DR5‐TRAIL apoptotic pathway. Subsequently, many more researchers have explored the possibility of enhancing anticancer drugs with EGCG and other catechins derived from green tea. Various combinations of EGCG and other anticancer drugs also showed synergistic anticancer effects on human cancer cell lines in both in culture and xenograft mouse models, and a mean reduction of 70.3% in tumor size was observed. On top of that, evidence has indicated that EGCG can inhibit the self‐renewal of cancer stem cells; thereby exhibiting a superior benefit when combined with other cancer drugs (Fujiki et al. 2015) (Table 4).

**TABLE 4:** Synergistic anticancer effects of catechins with conventional chemotherapy.

Combination	Cancer cell lines	Observed synergistic effects	Molecular mechanisms	References
EGCG + Sulindac	PC‐9 (lung)	↓ Cell growth ↑ Apoptosis	↑ p21 ↑ GADD153	Suganuma et al. (2011)
EGCG + Celecoxib	Human non‐small cell lung cancer	↑ Apoptosis	↑ MAPK ↑ GADD153	
Catechin + Chemotherapy agent	A549 Lung cancer cell	↓ Cell proliferation	↓ Cyclin E1 ↓ p‐Akt, ↑ p21, ↑ p27	Sun et al. (2020)
EGCG + Cisplatin	Biliary tract cancer cells	↑ Cell cycle arrest ↑ Apoptosis	↓ Expression of cell cycle genes ↑ DR5, ↑ p21	Mayr et al. (2015)
EGCG + NSAIDs	Animal tumor models	↓ Tumor growth	↑ GADD153‐DR5‐TRAIL ↑ Apoptosis	Fujiki et al. (2015)
EGCG + Various anticancer drugs	Various human cancer cell lines	Mean reduction of tumor size by 70.3%	↓ Cancer stem cell self‐renewal	

Abbreviations: DR5, death receptor 5; EGCG, epigallocatechin gallate; GADD153, growth arrest and DNA‐damage‐inducible gene 153; MAPK, mitogen‐activated protein kinase; NSAIDs, non‐steroidal anti‐inflammatory drugs; p21 and p27, cyclin‐dependent kinase inhibitors; p‐Akt, phosphorylated protein kinase B; TRAIL, TNF‐related apoptosis‐inducing ligand.

## 7 Therapeutic Perspectives

### 7.1 Other Medical Applications of Catechins

Tea catechins are being actively researched as an extension of the approach and are being employed more and more in science, medicine, and cosmetics (Bae et al. 2020). Most scientific research points to a connection between green tea and (−)‐EGCG with caries, microbes, periodontal disease, pulpal pathology, cancer, diabetes, colitis, and more. This connection is backed up with cell and animal model experiments as well as plenty of epidemiological and interventional studies in humans (Banerjee et al. 2021; Isemura et al. 2015). Catechin, as previously mentioned, is a powerful antioxidant, a property linked to cancer prevention and treatment, and is being examined in conjunction with various chemotherapeutic agents to boost their effectiveness and minimize drug‐related side effects (Shukla et al. 2018). In a study, daily drinking 10 cups of Japanese green tea and tablets of green tea extract declined the reappearance rate of colon polyps in people by half. Consequently, cancer patients who drink green tea together with anti‐cancer drugs are more secure. Nevertheless, it is not clear if the combination of GTC and cancer treatments might increase efficacy (Suganuma et al. 2011; Yu et al. 2014). A 0.5% catechin green tea was shown to prevent amyloid‐beta‐induced cognitive impairment in rats and reduce tau protein phosphorylation and inhibit NF‐κB activation. Additionally, when administered, EGCG improved mitochondrial respiration rate and membrane potential, ATP levels, as well as ROS production in A_PP/PS‐1 mice hippocampus, cortex, and striatum. The anti‐inflammatory effect of catechins, including EGCG, is linked to the presence of galloyl and hydroxyl moieties at the 3′ position. Also, catechins may also modulate pathways such as PKC, MAPK, and AKT which can be beneficial in AD pathophysiology (Banerjee et al. 2021). Catechins provide a multitude of health benefits, like antioxidant activity and reducing free radical damage and slowing the breakdown of the outer layers of cells due to ultraviolet radiation or contamination. The hydroxyls present in the gallate structure of EGCG and ECG are relatively more efficient at scavenging free radicals when compared to other antioxidants such as ascorbic acid, tocopherols, and Trolox. Due to their antioxidant and anti‐aging effects, green tea components, particularly catechins like EGCG, have been employed in the development of numerous medicinal and cosmetic formulations for topical and other uses. Oil‐in‐water emulsion model hydrogels, ointments, creams, and microsphere suspensions, muddy, liquid niosome, and ectosomes containing EGCG demonstrated effects on the photostability, skin penetration, skin elasticity, stratum corneum retention (bioavailability), and transepidermal water content and loss. In fact, catechins stimulate the production of collagen and prevent the development of matrix metalloproteinase enzymes, which immediately affect pores and skin. As well, a gelatin‐gamma‐polyglutamic acid ionic hydrogel containing EGCG demonstrated notable protection at a range of pH levels found in the environment of the gastrointestinal tract (Isemura et al. 2015). Tea catechins have been associated with improved cardiovascular health and lipid and glucose metabolism in obesity and Type 2 diabetes; these bioactive compounds aid in weight loss and may also reduce circulating lipid derivatives (Cerbin‐Koczorowska et al. 2021). By reducing oxidative stress and preventing platelet aggregation, catechins may also improve endothelial function (Muhammad and Dickinson 2019; Shukla et al. 2018). Polymers‐based catechin preparations such as EGCG‐loaded microspheres, Eudragit S100, the nonionic block copolymer Pluronic F127, or PLGA showed antihyperlipidemic and anti‐obesity activity (Isemura et al. 2015). Catechins show synergistic interactions with several antibiotics and enhance their efficacy against resistant microorganisms. Despite their many beneficial properties, catechins suffer from limited absorption and low bioavailability as effective therapeutics (Reygaert 2018; Wu and Brown 2021). A retrospective analysis of clinical data from 123 COVID‐19 patients identified three active traditional Chinese medicine (TCM) preparations. SARS‐CoV‐2 potent inhibitor of 3CLpro has been isolated from the active ingredients of TCM using a variety of methods, including surface plasmon resonance (SPR) binding assays, molecular docking, network pharmacology, and FRET‐based inhibition assays. The KD in SPR experiments was 6.17 M, suggesting that EGCG and 3CLpro interact well and have relatively high affinity for each other and for SARS‐CoV‐2 3CLpro (Reygaert 2018). Despite the fact that numerous scientific studies have uncovered the mechanisms by which catechins have positive effects on human health, there is still conflicting information regarding these substances' effects on people.

### 7.2 Challenges and Clinical Translation Gaps in the Anticancer Use of Catechins

Catechins, despite showing promising anticancer properties, face several limitations and clinical gaps that hinder their potential as therapeutic agents. One of the primary challenges is their low bioavailability; catechins are rapidly metabolized and eliminated from the body, which significantly reduces their effective concentration at target sites. This poor bioavailability is compounded by their instability in various physiological environments, leading to a rapid degradation and loss of therapeutic efficacy. Furthermore, the pharmacokinetics of catechins can be highly variable among individuals, influenced by factors such as gut microbiota composition, genetic variability in metabolism, and interactions with other dietary components or medications. Another limitation is the lack of comprehensive clinical trials to establish definitive therapeutic protocols, including optimal dosages and administration routes. Most of the current understanding of catechins' anticancer effects is derived from in vitro studies or animal models, which may not accurately represent their efficacy and safety in humans. Additionally, there is a need for more detailed mechanistic studies to fully understand how catechins interact at the molecular level within human cancer cells and the tumor microenvironment. Such insights are crucial for the development of more effective catechin‐based therapies and for identifying potential resistance mechanisms. The potential for adverse effects, particularly at higher doses or when used in combination with other treatments, also needs thorough investigation. While catechins are generally considered safe, their interaction with other anticancer agents could lead to unforeseen toxicities or reduced efficacy of conventional treatments. Moreover, the long‐term safety of catechins has not been extensively studied, especially in the context of chronic administration for cancer prevention or therapy. Lastly, there is a lack of standardized catechin formulations, which poses a challenge in comparing and replicating study results. The concentration, purity, and specific type of catechins used in research can vary widely, leading to inconsistencies in the data. Standardization is essential for advancing clinical research and for eventual integration into evidence‐based cancer treatment protocols. To overcome these barriers and fully harness the anticancer potential of catechins, future research should focus on enhancing their bioavailability, conducting rigorous clinical trials, exploring novel delivery systems, and developing standardized, high‐purity catechin formulations.

## 8 Conclusion

This comprehensive review has elucidated the intricate web of molecular pathways through which catechins, particularly (−)‐epigallocatechin‐3‐gallate (EGCG), exert anticancer effects. Our analyses confirm that catechins modulate key processes in carcinogenesis, including apoptosis, cell cycle arrest, and angiogenesis, and also exhibit the potential to inhibit metastasis. The mechanistic insights provided in this review strengthen the rationale for considering catechins as viable natural compounds in cancer therapeutics. However, despite promising in vitro and in vivo results, the translation of these findings into clinical practice remains limited by challenges such as bioavailability, stability, and the need for comprehensive human trials. Future investigations should aim to optimize the delivery and formulation of catechins, ensuring maximum therapeutic efficacy. Moreover, research should continue to explore the synergistic potential of catechins with existing anticancer drugs, with an emphasis on elucidating the molecular underpinnings of such interactions to advance toward clinical application.

## Author Contributions

**Patrick Valere Tsouh Fokou:** data curation (equal), investigation (equal), methodology (equal), validation (equal), visualization (equal), writing – original draft (equal), writing – review and editing (equal). **Boniface Kamdem Pone:** data curation (equal), investigation (equal), methodology (equal), writing – original draft (equal), writing – review and editing (equal). **Regina Appiah‐Oppong:** data curation (equal), investigation (equal), methodology (equal), writing – original draft (equal), writing – review and editing (equal). **Vincent Ngouana:** data curation (equal), investigation (equal), methodology (equal), writing – original draft (equal), writing – review and editing (equal). **Issakou Bakarnga‐Via:** data curation (equal), investigation (equal), methodology (equal), writing – original draft (equal), writing – review and editing (equal). **David Ntieche Woutouoba:** data curation (equal), investigation (equal), methodology (equal), writing – original draft (equal), writing – review and editing (equal). **Valerie Flore Donfack Donkeng:** data curation (equal), investigation (equal), methodology (equal), writing – original draft (equal), writing – review and editing (equal). **Lauve Rachel Tchokouaha Yamthe:** data curation (equal), investigation (equal), methodology (equal), writing – original draft (equal), writing – review and editing (equal). **Fabrice Fekam Boyom:** data curation (equal), investigation (equal), methodology (equal), writing – original draft (equal), writing – review and editing (equal). **Dilek Arslan Ateşşahin:** data curation (equal), investigation (equal), methodology (equal), writing – original draft (equal), writing – review and editing (equal). **Javad Sharifi‐Rad:** conceptualization (equal), data curation (equal), investigation (equal), methodology (equal), project administration (equal), supervision (equal), validation (equal), visualization (equal), writing – original draft (equal), writing – review and editing (equal). **Daniela Calina:** data curation (equal), investigation (equal), methodology (equal), supervision (equal), validation (equal), visualization (equal), writing – original draft (equal), writing – review and editing (equal).

## Conflicts of Interest

The authors declare no conflicts of interest.

## Data Availability

The authors have nothing to report.
